# Recurrent Incisional Hernia Repair—An Overview

**DOI:** 10.3389/fsurg.2019.00026

**Published:** 2019-05-14

**Authors:** Ferdinand Köckerling

**Affiliations:** Department of Surgery and Center for Minimally Invasive Surgery, Academic Teaching Hospital of Charité Medical School, Vivantes Hospital, Berlin, Germany

**Keywords:** recurrent incisional hernia, laparoscopic IPOM, sublay, component separation technique, open IPOM

## Abstract

**Introduction:** Recurrent incisional hernias with a rate of around 20% account for a relatively large proportion of all incisional hernias. It is difficult to issue any binding recommendations on optimum treatment in view of the relatively few studies available on this topic. This review now aims to collate the data available on recurrent incisional hernia.

**Material and Methods:** A systematic search of the available literature was performed in January 2019 using Medline, PubMed, Scopus, Embase, Springer Link, and the Cochrane Library, as well as a search of relevant journals and reference lists. For the present analysis, 47 publications were identified as relevant.

**Results:** There are mainly case series available on the treatment of recurrent incisional hernia. Eight evaluable case series and two prospective comparative studies report on treatment of between 27 and 85 recurrent hernias. After primary open repair of incisional hernia and defect sizes of < 8–10 cm, the recurrence operation can be performed in laparoscopic technique provided the surgeon has sufficient experience in that procedure. That also applies to multiple recurrences after exclusively open repair. There are no evaluable data on a repeat laparoscopic approach after minimally invasive repair of primary incisional hernia. Such an approach should only be chosen by very experienced laparoscopic surgeons and based on a well-founded indication. Further data are urgently needed on treatment of recurrent incisional hernia.

**Conclusion:** Very little data are available on the treatment of recurrent incisional hernia. Based on the tailored approach concept, a laparoscopic approach undertaken by an experienced laparoscopic surgeon can be recommended for recurrent hernias after primary open repair and for defects of up to 8–10 cm.

## Introduction

All guidelines on the treatment of incisional hernias recommend, as Grade A recommendation based on the Oxford criteria of evidence-based medicine, the use of meshes (1–7). But even when using meshes recurrence rates of up to between 25 and 32% are observed after 5 and 10 years (8–10). It is only after 10 years' follow-up that the actual recurrence rate can be estimated (11). Recurrence of an incisional hernia constitutes an unfavorable prognostic factor based on the classification of primary and incisional abdominal wall hernias of the European Hernia Society (12). Recurrence of an incisional hernia after previous mesh repair meets the criteria for a complex abdominal wall hernia (13). “Recurrent incisional hernia repairs are technically difficult operations for many reasons: mesh has been placed, there is potential for dense adhesion to the abdominal wall and anatomical planes have been disturbed by previous dissection” (14). Although registry analyses have revealed that recurrent incisional hernias account for a 22% proportion of all incisional hernia repairs, which are routine procedures in everyday hernia surgery (11), to date there is a paucity of publications on this topic. Therefore, this review of the literature now aims to collate and evaluate those studies available.

## Materials and Methods

**Figure 1:**
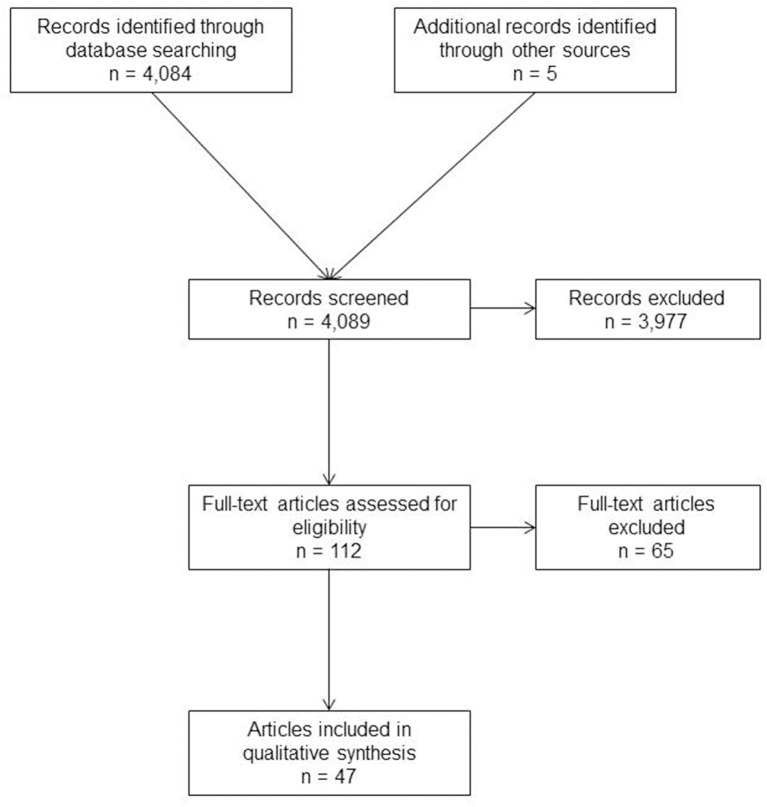
Prisma flow diagram of study inclusion.

A systematic search of the available literature was performed in January 2019 using Medline, PubMed, Scopus, Embase, Springer Link, and the Cochrane Library, as well as a search of relevant journals and reference lists. The following search terms were used: “incisional hernia,” “recurrent incisional hernia,” “incisional hernia and recurrence,” “recurrent incisional hernia repair” and “recurrent hernia.” The title and abstracts of 4,089 publications were screened (Figure 1).

For the present analysis, 47 publications were identified as relevant for the key question. A systematic presentation and synthesis of the characteristics and findings of the included studies have been undertaken in accordance with the Prisma guidelines (15). Since there are significant differences in the outcomes of primary and incisional ventral hernia repairs, only studies that provide clear insights into the outcome of incisional hernia repair are included (16–20).

## Recurrence Rates According to the Techniques in Primary Incisional Hernia Repair

The guidelines on primary and incisional ventral hernia repair recommend, as an evidence-based recommendation,” the laparoscopic intraperitoneal onlay mesh (IPOM) and open sublay techniques as the best techniques for repair of an incisional hernia” (1–7). But the guidelines also see a role for other positions of mesh placements (open IPOM, onlay) (1). For complex abdominal wall hernias the perforator-sparing, endoscopic and posterior component separation techniques are recommended (1).

## Laparoscopic IPOM

In a meta-analysis of six randomized controlled trials (RCTs) with a total of 366 patients, the recurrence rate after laparoscopic IPOM of incisional hernia at follow-up of 2–35 months was 8.7% (21).

Analysis of data from the Danish Hernia Database between January 1, 2007 and December 31, 2010 identified a reoperation rate for recurrence of 15.5% at median follow-up of 21 months after elective laparoscopic IPOM incisional hernia repair (22). This nationwide study included 1.763 laparoscopic repairs with a conversion rate of 7.1%. Laparoscopic incisional hernia repairs were performed by 32 departments with a median number of 57 cases (range 12–78) (22). The follow-up rate was 100% (22).

A further but recent analysis of data from the Danish Hernia Database revealed at median follow-up of 61 months with a follow-up rate of 100% a need for repair of recurrent hernia after 1.757 laparoscopic IPOM in 10.6% of cases (23).

The recurrence rates were considerably higher in the Danish Hernia Database when the reoperation rate due to recurrence was supplemented by patient questioning and clinical examination, and CT when indicated (24). Depending on whether an absorbable or non-absorbable tacker was used for mesh fixation in laparoscopic IPOM, recurrence rates of 28.5 and 18.0%, respectively, were identified at median follow-up of 40 months (24). 816 of 1.037 patients (follow-up rate 78.7%) were included in the analysis. 174 patients with suspicion of recurrence were examined.

In a propensity score-matched observational, single-center study of incisional hernia repair in laparoscopic vs. open IPOM with a mean follow-up of 5.5 years, the recurrence rate for the laparoscopic approach was 20% (25).

## Open Sublay

In a review article of sublay repair in incisional hernia a mean recurrence rate of 13.5% (range 1.6–32.0%) was identified at follow-up of 1 to 10 years (26). In this review article all available studies comparing sublay with other incisional hernia repair techniques were collected and the outcome observed. Only 9 randomized controlled trials and 2 comparative registry studies could be analyzed for the recurrence rate following incisional hernia repair in sublay technique.

## Open IPOM

For the open IPOM technique in incisional hernia repair a review revealed a recurrence rate of 12.6% (range 0–61%) at follow-up of 1–8.1 years (27). These outcome data based mainly on prospective and retrospective observational studies. Defect closure appears to have a positive influence on the recurrence rate.

## Open Onlay

In another review of the onlay technique for incisional hernia repair the mean recurrence rate was 9.9%, with a range of 0–32% at follow-up of 1–8 years (28). Five randomized controlled trials and 17 observational studies were available for this analysis of the recurrence rate following incisional hernia repair in onlay technique. Defect closure reduces the recurrence rate.

## Posterior Component Separation Technique—Transversus Abdominis Release

In a systematic review the mean recurrence rate in the transversus abdominis release for incisional hernia repair at follow-up of 7–50 months demonstrated a low mean recurrence rate of 5.25% (29). Only prospective and retrospective observational studies were available for this analysis. The degree of contamination ranged from 0 to 92%.

## Percentage of Recurrent Hernias of the Total Incisional Hernia Patient Population

In the Herniamed Hernia Registry, 21.85% (*n* = 5,328) of all incisional hernias (*n* = 24,385) were recurrent incisional hernias (11). In the Danish Hernia Database, 18.2% (*n* = 593) of 3,258 elective incisional hernia repairs had a previous incisional hernia operation (22). Hence, for every one in five patients undergoing elective incisional hernia repair, the operation involved a recurrence operation. However, that only reflects the proportion of patients who actually submit to reoperation because of incisional hernia recurrence. But since not all patients with recurrent incisional hernia undergo reoperation, the proportion is even higher. In a follow-up-study based on data from the Danish Hernia Database the recurrence rate after elective, primary incisional hernia repair was as high as 37% (median follow-up of 41 months; range 0–48 months) (30). The median time interval between primary incisional hernia and recurrent repair was 12 month (30).

## Arguments Pro Laparoscopic Repair of Recurrent Incisional Hernias

“Laparoscopic incisional hernia repair has been shown to have decreased wound events, decreased postoperative pain and overall decreased length of stay compared to an open approach” (2–7, 14).

The updated guidelines of the European Association of Endoscopic Surgery (EAES) and the European Hernia Society (EHS) state that, as a strong recommendation (Panel Consensus 100%), “incisional hernia recurrence can be treated by laparoscopy either after primary open or laparoscopic surgery without the need for mesh removal” (3). That statement has been confirmed by literature data, but no specific data on the outcome of the subgroup of recurrent incisional hernias and the previous incisional hernia repairs have been reported (31–34). The proportion of recurrence operations in laparoscopic technique for incisional hernias in that series was up to 28% (31–34), for ventral hernias up to 34% (35).

The guidelines of the International Endohernia Society (IEHS) for laparoscopic treatment of ventral and incisional abdominal wall hernias recommend for recurrence after previous open incisional hernia repair laparoscopic management “provided the surgeon has sufficient experience in laparoscopic incisional hernia repair” (4). “After open mesh repair, reoperation by the laparoscopic approach has certain advantages” (4). “First, the reoperation is performed at a different site / level of the abdominal wall” (4). “Second, in all instances, the entire incisional scar can be covered by a mesh” (4). “Usually it is not necessary to remove the previously inserted mesh, hence avoiding extensive dissection of the abdominal wall” (4). “A further possible advantage of a laparoscopic reoperation is the identification of previously undiscovered, occult incisional hernias” (4).

In a series of 1,242 laparoscopic ventral/incisional hernia repairs over a period of 13 years occult hernias were observed in 16.3% of the patients (36).

## Case Series of Laparoscopic Recurrent Incisional Hernia Repair

The case records of 69 patients with recurrent incisional hernia who underwent laparoscopic repair were reviewed by Ferrari et al. (37) (Table 1). The previous repairs have been performed in seven patients by laparoscopy, in 41 patients by prosthetic open technique and in 36 patients by suture (37). “No conversion occurred, but five intraoperative complications (7.2%) were recorded: three bowel injuries, one omentum bleeding and one epigastric vessel lesion” (37). “Postoperative mortality was null, while overall morbidity was 13% (nine patients) with a prevalence of seroma lasting over 8 weeks in six patients (8.7%).” “With a mean follow-up of 41 months (range 6–119), the recurrence rate was 5.7% (four patients).” The authors concluded that surgical treatment for recurrent incisional hernia remained controversial because of the paucity of literature reporting on specific studies for this topic (37). “Morbid obesity and large defects have often been associated with technical difficulties and worse results” (37). Patients with a BMI >30 had a larger defect, longer operation time and length of hospital stay (37). There was no difference in the postoperative complication rate.

**Table 1:** Laparoscopic recurrent incisional hernia repair.

**Refereneces**	**Operative technique**	**Previous repair**	**n**	**Study type**	**Intraoperative complications**	**Postoperative complications**	**Mortality**	**Follow-up**	**Recurrence rate**	**Defect closure**	**Mesh overlap**	**Mesh type**
Ferrari et al. (37)	Lap IPOM	Suture, open mesh, lap IPOM	69	Case series	7.2% (3 bowel injuries, 1 omental bleeding, 1 epigastric vessel bleeding)	13% (including 6 seromas = 8.7%)	0%	Mean 41 months, range 6–119 months)	5.7%	Not reported	5 cm	ePTFE
McKinlay and Park (38)	Lap IPOM	—	69	Prospective comparative study	2.9% (2 bowel injuries)	28%	—	Mean 19 ± 18 months	7%	Not reported	3–5 cm	ePTFE
Verbo et al. (39)	Lap IPOM	Suture, open mesh	41	Case series	—	17% (including 3 seromas = 7.3%)	—	Mean 38 months, range 18–54	2.4%	Not reported	—	ePTFE
Meyer et al. (40)	Lap IPOM	—	34	Prospective comparative study	—	Major: 17.6%	—	Median 942 days, range 399-1693	26.5%	Not reported	5 cm	Composite polyester mesh
Uranues et al. (41)	Lap IPOM	Suture, open mesh	85	Case series	Conversion 1.2%	15.2% (including port-site cellulitis 1%, seroma 7%, persistant pain 7%)	—	Median 41 months, range 24–61	3.5%	Not reported	5 cm	ePTFE
Picazo-Yeste et al. (42)	Lap IPOM	Suture, open mesh	71	case series	Conversion 2.8%	12.7%	—	Mean 30 months, range 20–90	1.4%	Not reported	5 cm	Composite polypropylene

In a prospective observational study by McKinlay and Park (38) patients with recurrent incisional hernia (*n* = 69) were compared to patients with primary incisional hernia (*n* = 101) (Table 1). In this study the authors evaluated the efficiency of repairing recurrent incisional hernia laparoscopically (38). The patients with recurrent incisional hernia had a mean of 1.9 ± 1.3 previous repairs, higher body mass index and larger defect size. “The complication rate was higher in the recurrent group (28 vs. 11%, p = 0.001), but the recurrence rate was not different (7 vs. 5%, *p* = 0.53)” (38). “The authors concluded that laparoscopic repair of recurrent incisional hernia was an effective alternative to conventional repair” (38).

In a prospective case series reported by Verbo et al. (39) 41 consecutive patients with recurrent incisional hernias were treated laparoscopically (Table 1). Twenty-six patients suffered from the first recurrence, whereas the other 15 had undergone multiple prior repairs. In all cases, the prior repairs were performed by the traditional open approach, with mesh positioning in 27 cases and suture repair in the remaining 14 (39). Twelve patients had their first recurrence after incisional hernia repair, eight their second recurrence, five their third recurrence and two their fourth recurrence. Complications were reported in 17% of cases including two cases with prolonged ileus, one urinary tract infection, two seromas, one case of prolonged (>24 h) postoperative pain and one major complication with enterotomy (39). Only one recurrence (2.4%) was reported for a mean follow-up of 38 months (range: 18–54 months) (39).

“The authors concluded that laparoscopic repair of recurrent incisional hernia seemed to be an effective alternative to the conventional approach since it could result in lower recurrence and complication rates” (39).

In a comparative study on laparoscopic treatment of 64 primary vs. 34 recurrent incisional hernia significantly more major complications were documented (12.5 vs. 17.6%) for the recurrent incisional hernia repair (40) (Table 1). With a median follow-up of 942 days (range 339–1693 days) the recurrence rate for primary incisional hernia repair was 11.0 vs. 26.5% for recurrent incisional hernia (40). “The authors concluded that laparoscopic treatment of recurrent incisional hernias was associated with high rates of minor and major complications (40). Therefore, the surgeon needs to have a high level of expertise” (40).

Uranues et al. (41) demonstrated in a retrospective observational study of 85 patients with recurrent incisional hernia repair in laparoscopic technique a low conversion rate (*n* = 1/85; 1.2%) and a 15.2% adverse event rate, including 1% port-site cellulitis, 7% seroma, and 7% persistent pain (41) (Table 1). The hernia recurrence rate was 3.5% at 41-months' (range 24 to 61 months) follow-up (41). “The authors concluded that laparoscopic recurrent incisional hernia repair resulted in a low rate of adverse events and a risk of recurrence similar to the rates associated with first-time hernia repair” (41).

Picazo-Yeste et al. (42) presented a consecutive case series of 71 recurrent incisional hernia repairs (Table 1). The conversion rate was 2.8%, the postoperative complication rate 12.7% and the recurrence rate for a mean follow-up of 30 months (range 20–90 months) was 1.4% (42).

## Limitations of Laparoscopic Recurrent Incisional Hernia Repair

The findings presented above on laparoscopic repair of recurrent incisional hernias demonstrate that, if the surgeon has the necessary expertise, good results can be obtained but there is still a high risk of intra- and postoperative complications. There appears to be a much higher incidence of intraoperative intestinal injury and of postoperative seroma compared with laparoscopic repair of primary incisional hernias. Morbid obesity and large defects are obvious limiting factors. In the guidelines on laparoscopic treatment of ventral/incisional hernias a defect size of 8–10 cm is given as the limit for this indication (2–4). While no study has investigated this in the case of recurrent incisional hernias, that limit is also likely to apply here. Since the proportion of patients with previous laparoscopic repair of primary incisional hernia is small, this indication makes high demands on the surgeon since markedly more adhesions to the intestines are expected than after conventional primary repair. The mesh size used for the previous laparoscopic repair of primary incisional hernia will no doubt play a role in the indication. The larger the mesh used, the greater is the likelihood of extensive adhesions to the intestines, but sometimes only parts of the implanted mesh show adhesions, especially when covered polypropylene meshes were used and the defects were closed. Therefore, the patient's BMI, the defect size and the primary incisional hernia repair technique should be taken into account when indicating a laparoscopic approach for recurrent incisional hernia repair. A primary prerequisite is that the surgeon should have vast experience in the laparoscopic IPOM technique. As such, the indication for laparoscopic recurrent incisional hernia repair must always be considered on a case by case basis while taking account of patient factors and of details of the previous primary repair. The experience of the surgeon is hard to define, because no data on the learning curve for laparoscopic recurrent incisional hernia repair are available.

## Arguments Pro Open Repair of Recurrent Incisional Hernias

“Factors that lead to recurrent incisional hernias often necessitate open repair” (14). “For instance, patients with large or multiple defects, significant intra-abdominal adhesions, or compromised, often overlying, skin integrity may require an open approach” (14).

## Case Series of Open Recurrent Incisional Hernia Repair

Berry et al. (43) presented the data of 47 recurrent incisional hernia repairs in retromuscular (sublay) technique and panniculectomy. The 13 male and 34 female patients with an average body mass index of 34.4 kg/m^2^, an average midline hernia defect of 31.4 cm, and at least 1 and on average 2.5 previous repair attempts underwent sublay recurrent incisional hernia repair with the use of different meshes (43) (Table 2). Wound infection occurred in four patients (8%) and seroma requiring aspiration occurred in one patient (2%). Four patients (8%) had re-recurrences of their hernias in a mean follow-up of 608 ± 74 days (42). “All patients rated the postoperative appearance of their abdomen as at least satisfactory.” “The authors concluded that recurrent incisional hernia repair with a retromuscular (sublay) mesh and panniculectomy had low recurrence and wound complication rates as well as excellent patient satisfaction” (43).

**Table 2:** Open recurrent incisional hernia repair.

**Refereneces**	**Operative technique**	**Previous repair**	**n**	**Study type**	**Intraoperative complications**	**Postoperative complications**	**Mortality**	**Follow-up**	**Recurrence rate**	**Mesh type**
Berry et al. (43)	Retromuscular = sublay repair + panniculectomy	—	47	Case series	—	Thromboembolic event 13%, wound infection 8%, wound dehiscense 6%	—	Mean 608 ± 74 days	8%	ePTFE, Polypropylene and Polyglactin 910, Polypropylene
Temudom et al. (44)	Modified Stoppa technique	—	27	Case series	—	Wound infection 8%, mesh removal 2%	—	Mean 24 months, range 2–56 months	0%	ePTFE, Polypropylene
Novitsky et al. (45)	Preperitoneal retrofascial mesh repair	—	32	Case series	—	Wound infection 12.5%	—	Mean 28.1 months, range 8–60	3.1%	Polypropylene
DiBello and Moore (46)	Compound flap of the rectus muscle	—	35	Case series	—	Seroma 2.8%, wound infection 5.7%, hematoma 5.7%	—	Mean 22 months, range 1–43	8.5%	ePTFE, Polypropylene and Polyglactin 910

Temudom et al. (44) reported on their experience with 50 patients suffering from complex giant or recurrent incisional hernias (Table 2). Twenty-seven patients had undergone one to five previous hernia repairs. The mean follow-up (100%) was 24 months. “They used a modified Stoppa technique placing a very large sheet of polypropylene mesh in the plane anterior to the posterior rectus fascia but posterior to the rectus muscle” (44).

“Because the mesh will patch the defects between the medial edges of the posterior rectus fascia in a tension-free manner, the entire peritoneal sac should be preserved to serve as a barrier between the posterior surface of the mesh and the intraperitoneal contents” (44). Wound infection occurred in four patients (8%). Two patients needed mesh removal (4%). For the remaining 48 patients the recurrence rate for a mean follow-up of 24 months (range 2–56 months) was null (44).

Novitsky et al. (45) “conducted a retrospective review of consecutive patients undergoing open preperitoneal retrofascial mesh repair of multiply (two or more) recurrent hernias” (Table 2). “A preperitoneal plane was entered, and peritoneal flaps were developed circumferentially” (45). Thirty-two patients with multiple incisional hernias underwent surgical repair (45). “The number of previous herniorrhaphies was 3.6 (range 2 to 24)” (45). There were no major intraoperative complications. Wound infection occurred in four patients (12.5%). With a mean follow-up of 28.1 months (range 8 to 60 months) there was recurrence (3.1%) (45). “The authors concluded that open preperitoneal retrofascial mesh repair resulted in an effective herniorrhaphy with low perioperative morbidity” (45).

DiBello and Moore (46) reported on 35 patients with recurrent incisional hernia treated by a “compound flap of the rectus muscle with its attached internal oblique—transversus abdominis muscle with advancement to the midline to recreate the linea alba” (Table 2). The overall recurrence rate was 8.5% (*n* = 3/35) for a mean follow-up of 22 months (range 1–43 months) (46). “Additional complications, namely seroma, wound infection, and hematoma, occurred with rates of 2.8, 5.7, and 5.7 percent, respectively” (46).

In a study by Hultman et al. (47) 16 patients with a recurrence after component separation of incisional hernias underwent secondary repair of a recurrence. Of these 16 recurrences, 15 had successful repair (46). “Successful repair of these recurrent hernias was achieved by placement of additional mesh in 14 of 15 patients and primary fascial closure in one patient” (47).

## Discussion

Although recurrent incisional hernias with a rate of around 20% account for a relatively large proportion of all incisional hernias, to date very few studies have been published on their treatment. In particular, there is a lack of comparative studies on open vs. laparoscopic recurrent incisional hernia repair.

Nonetheless, in the updated guidelines of the European Association of Endoscopic Surgery (EAES) and the European Hernia Society (EHS) state with a panel consensus of 100%, as a strong recommendation, that laparoscopic repair of incisional hernias after previous primary open and laparoscopic repairs should be used (3). Conversely, the Guidelines of the International Endohernia Society (IEHS) tend to recommend laparoscopic repair of recurrent incisional hernia after previous primary open repair (4). The principle arguments in favor of a laparoscopic approach after primary open incisional hernia repair are that reoperations are performed in a different anatomic layer, the entire incisional scar can be covered (by a mesh), avoidance of extensive dissection of the abdominal wall by not having to remove the previously inserted mesh, and the ability to diagnose occult incisional hernias (4).

The literature does not have any definitive or precise data on a laparoscopic approach after previous primary laparoscopic repair of incisional hernia. Therefore, any indication for such an approach must be subjected to critical scrutiny. To that effect, corresponding data are urgently needed before such an approach can be recommended in the guidelines.

Those studies available on laparoscopic recurrent incisional hernia repair after previous open incisional hernia repair with and without mesh (37–42) reveal that such an approach can be used with good outcomes provided that the surgeon has the necessary experience. Nonetheless, the available studies demonstrate a markedly higher risk of intraoperative (bowel injuries) and postoperative complications (seromas). Likewise, the recurrence rates have a considerably broad range of 1.4–26.5% (37–42). While there are no data to that effect, it can be assumed that just as in the case of primary incisional hernias, for recurrence too the outcomes achieved for defects >8–10 cm would be poorer (1–7). Therefore, extreme caution should be exercised if indicating a laparoscopic operation for recurrent hernia repair after previous open repair for defects >8–10 cm, even though at present there are no data on laparoscopic repair of recurrent incisional herma. Thus, using the tailored approach concept, a laparoscopic approach can be indicated for recurrent incisional hernia repair if the primary repair was performed as an open technique with or without mesh and the defect size measured on computed tomography or magnetic resonance imaging was not >8–10 cm. For larger defects and when a laparoscopic IPOM operation was used for primary incisional hernia repair, recurrent incisional hernia repair should in general be performed in open technique. Only a very experienced laparoscopic surgeon can contemplate using once again a laparoscopic approach for a recurrence after previous laparoscopic repair of a primary incisional hernia. Further studies are urgently needed in order in the future to gain better insights into the optimum approach for recurrent incisional hernias. These studies should, in particular, investigate whether the laparoscopic technique is also suitable for recurrent incisional hernia after previous primary incisional hernia repair in laparoscopic technique and also whether on using laparoscopic repair for recurrent incisional hernias with defects >8–10 cm significantly poorer results would have to be expected. Additionally, no data are available on the role of defect closure, optimal mesh overlap, best fixation technique, and preferable mesh type in laparoscopic treatment of recurrent incisional hernia after previous open mesh repair. Also the necessary experience of a surgeon for the laparoscopic repair of a recurrent incisional hernia is under debate, because no data on this topic are existing. A surgeon performing laparoscopic recurrent incisional hernia repair should at least have a vast experience with laparoscopic primary incisional hernia repair.

## Summary

It can be stated that 20% of all incisional hernia repairs involve recurrent procedures and hence one in every five patients with incisional hernia experiences a recurrence. After previous primary open incisional hernia repair and defect sizes of 8–10 cm, the laparoscopic technique is a good option but calls for an experienced laparoscopic surgeon. Intraoperative intestinal injuries and postoperative seromas present a particular risk. The role of laparoscopic reoperations after previous primary incisional hernia repair in laparoscopic IPOM technique has not been clarified to date. Likewise, there is little information on the open approach for recurrent incisional hernias. In view of the high incidence of recurrent incisional hernias further studies are urgently needed.

## Recommendation

The limited number and quality of the existing studies on recurrent incisional hernia repair do not allow any strong recommendation. Further studies with comparison of the laparoscopic vs. open approach in recurrent incisional hernia repair are urgently needed.

## Author Contributions

FK: literature search, literature analysis, publication concept, and publication draft.

### Conflict of Interest Statement

The author declares that the research was conducted in the absence of any commercial or financial relationships that could be construed as a potential conflict of interest.
